# Two Rare Cases of Metastatic Occult Thyroid Carcinoma Without Primary Cancer in the Thyroid Gland

**DOI:** 10.7759/cureus.63280

**Published:** 2024-06-27

**Authors:** Kenny H Do, Emily S Sagalow, Richard Wang, Oluwafunmilola T Okuyemi, Jo-Lawrence Bigcas

**Affiliations:** 1 Department of Otolaryngology – Head and Neck Surgery, Kirk Kerkorian School of Medicine at University of Nevada, Las Vegas (UNLV), Las Vegas, USA

**Keywords:** head and neck surgery, otolaryngology, thyroid metastasis, neck dissection, papillary thyroid carcinoma, occult thyroid carcinoma

## Abstract

Papillary thyroid cancer (PTC) contributes to the majority of all thyroid malignancies. In this case report, we detail two cases of occult thyroid carcinoma (OTC), which presents with thyroid metastasis to locoregional lymph nodes without having an initial primary tumor detected in the thyroid gland. OTC may be found incidentally on biopsy, surgery, or imaging. Advancements in diagnostic technology have allowed physicians to identify and treat OTC at an earlier stage. We present two patients who were found to have metastases to cervical lymph nodes without a primary identification in the thyroid gland.

The first patient was a 67-year-old female who noticed an enlarging mass in her right neck at levels III and IV. Fine needle aspiration (FNA) revealed the presence of PTC. The patient underwent a total thyroidectomy, central nodal dissection, and right-modified radical neck dissection. Final pathology confirmed the presence of PTC metastasis to cervical lymph nodes, but no primary tumor was identified within the thyroid gland.

The second patient was a 79-year-old male who presented with a painless mass of the left parotid gland. The FNA of the patient revealed PTC metastasis to his left parotid gland. The patient underwent a total thyroidectomy, ipsilateral central nodal dissection, ipsilateral modified radical neck dissection, and inferior superficial and deep lobe parotidectomy. No malignancy was detected within the thyroid gland or central or lateral neck lymph nodes on final pathology. Carcinoma was confined to an intra-parotid node in the deep lobe of the parotid gland.

OTC is a rare phenomenon in PTC. One proposed theory for OTC includes spontaneous regression of the primary tumor and genetic mutations to the BRAF gene. Due to the fact that it is easy for this rare condition to be misdiagnosed, more studies should be conducted to standardize diagnostic and treatment plans for OTC.

## Introduction

The incidence of papillary thyroid cancer (PTC) has been steadily increasing over recent years, with the disease now comprising up to 80% of all thyroid malignancies [1,2]. Generally speaking, it is rare for cancer to metastasize to locoregional lymph nodes when there is no primary tumor detected in the thyroid gland [1,2]. This phenomenon is known as occult thyroid carcinoma (OTC), defined as the presence of a benign thyroid gland on histology even though there is evidence of metastatic thyroid carcinoma within extrathyroidal sites [1,3]. Due to its unusual nature, OTC is often only detected incidentally during surgery, biopsy, autopsy, and imaging [4].

Usually, in OTC, thorough testing reveals papillary microcarcinomas less than 1 cm in size [1]. Liu et al. and Boucek et al. created five classifications for OTC. The initial subtype encompasses the identification of OTC subsequent to a thyroidectomy for a non-malignant condition. The second subtype involves the incidental discovery of OTC through a fine needle biopsy or ultrasound examination. The third subtype is characterized by thyroid metastasis that remains undetected prior to surgery but is later revealed under histology. The fourth subtype pertains to cases with ectopic thyroid tissue [4,5]. According to these classifications, it is rare for the fifth category to occur, which involves metastasis to lymph nodes and organs in the absence of cancer in the thyroid gland [4,5].

One study suggests that the prevalence of the fifth OTC classification is around 0.3%, while another suggests that it is around 5-10% in the general population. While the specific metastatic sites for patients with OTC remain uncertain due to its rarity, individuals with PTC typically experience metastasis most frequently to the cervical lymph nodes situated in the central compartment of the neck [6-8]. The progress in diagnostic technology within otolaryngology, including ultrasonography-guided fine needle aspiration (FNA) biopsy, has enhanced the accuracy and detectability of OTC diagnosis [6]. This case report details two patients who presented with thyroid cancer metastases in the absence of primary thyroid cancer detected on final pathology, with one experiencing metastasis to the cervical lymph nodes and another experiencing metastasis to the parotid gland.

## Case presentation

Patient 1

A 67-year-old female patient with a prior history of atrial fibrillation on Apixaban presented to the otolaryngology clinic for evaluation of progressively enlarging right-sided levels III and IV cervical adenopathy. She reported dysphagia but otherwise denied a history of fever, pain, dyspnea, weight loss, or dysphonia.

**Figure 1:**
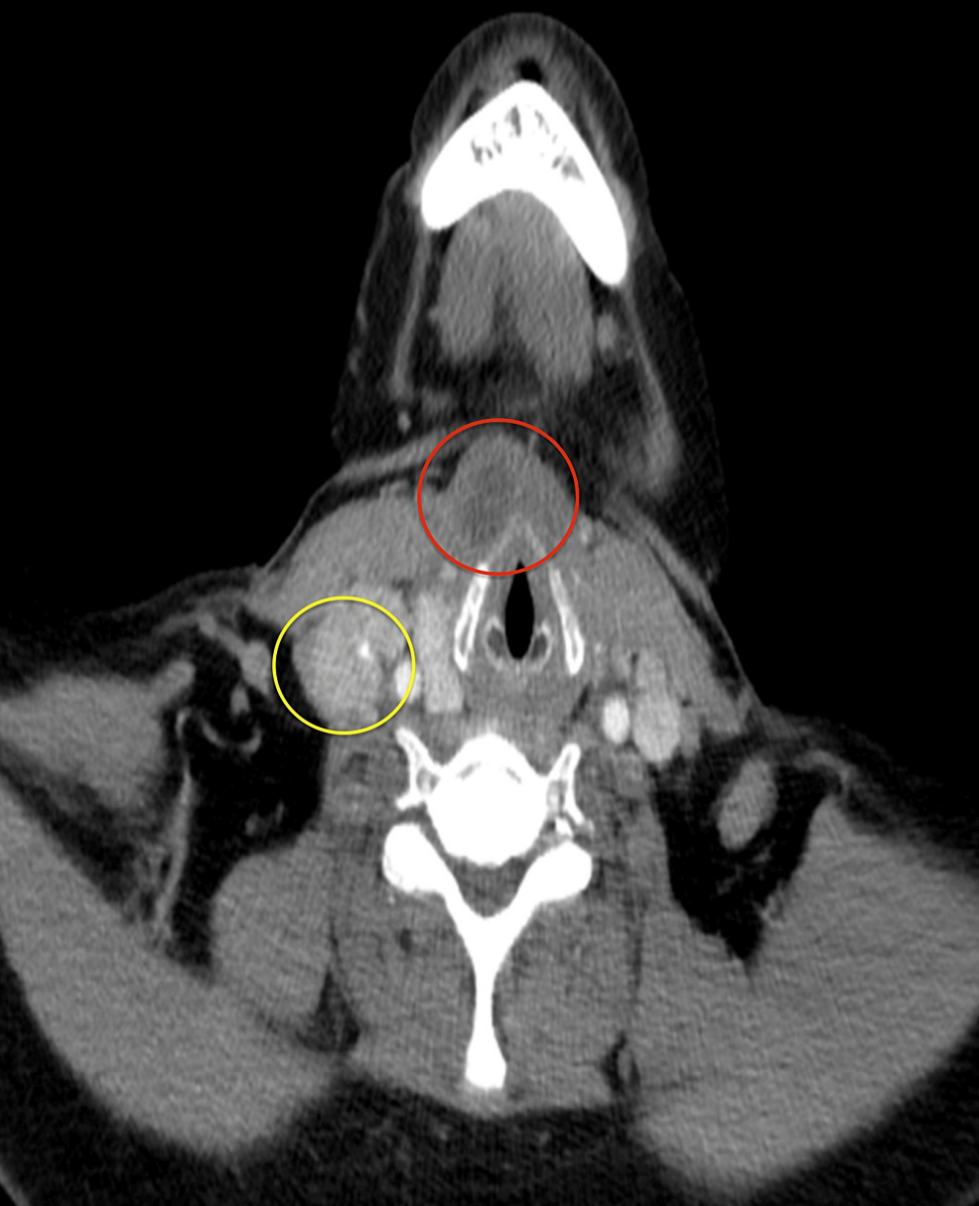
Axial-cut CT neck with contrast demonstrating both right lateral neck metastatic lymph node in levels III/IV and a prelaryngeal mass in the area of the pyramidal lobe for Patient 1 Yellow circle: metastatic lymphadenopathy in levels III/IV, red circle: thyroglossal duct cyst CT: computed tomography

A physical examination revealed painless and firm lymphadenopathy of the right lateral neck. A CT scan (Figure 1) demonstrates right-sided cervical adenopathy lateral to the jugular vein, revealing metastatic lymph nodes on the right side of levels III/IV, along with a prelaryngeal lesion near the pyramidal lobe. One consideration for that finding was a possible thyroglossal ductal cyst. Subsequent soft tissue ultrasound also demonstrated right lateral neck adenopathy. The FNA of the right lateral neck lymph node was consistent with the PTC. A dedicated thyroid ultrasound demonstrated two nodules. The first nodule was graded as Thyroid Imaging, Reporting, and Data System (TI-RADS) 3: 1.3 cm solid, well-circumscribed, hyperechoic nodule located in the right inferior thyroid lobe, noted to be wider than tall without internal calcifications. The second nodule was graded as TI-RADS 2: 1.1 cm mixed solid and cystic architecture located within the isthmus, smooth margins, a wider-than-tall configuration, and being isoechoic. There was no evidence of metastatic spread to the left neck lymph nodes on imaging. Given the histology and locoregional metastasis, surgery was recommended, including total thyroidectomy with right central neck dissection and right modified radical neck dissection. FNA of the thyroid was considered unnecessary due to the presence of disease in the lateral neck and the patient's indication for thyroidectomy and radioactive iodine treatment. Initially, it was assumed that the primary cancer would be in the thyroid, rendering FNA redundant in this instance. The sistrunk procedure was also considered if thyroglossal ductal carcinoma was identified intraoperatively.

The patient underwent total thyroidectomy, central nodal dissection, and right lateral neck dissection levels II through IV. Thyroidectomy and central nodal dissection were largely unremarkable. Parathyroid reimplantation of the right-sided parathyroids was performed. The pyramidal lobe was found to be benign, and central hyoidectomy was withheld. Aside from the complete unraveling of the carotid sheath in levels III and IV, the right lateral neck dissection was also unremarkable.

**Figure 2:**
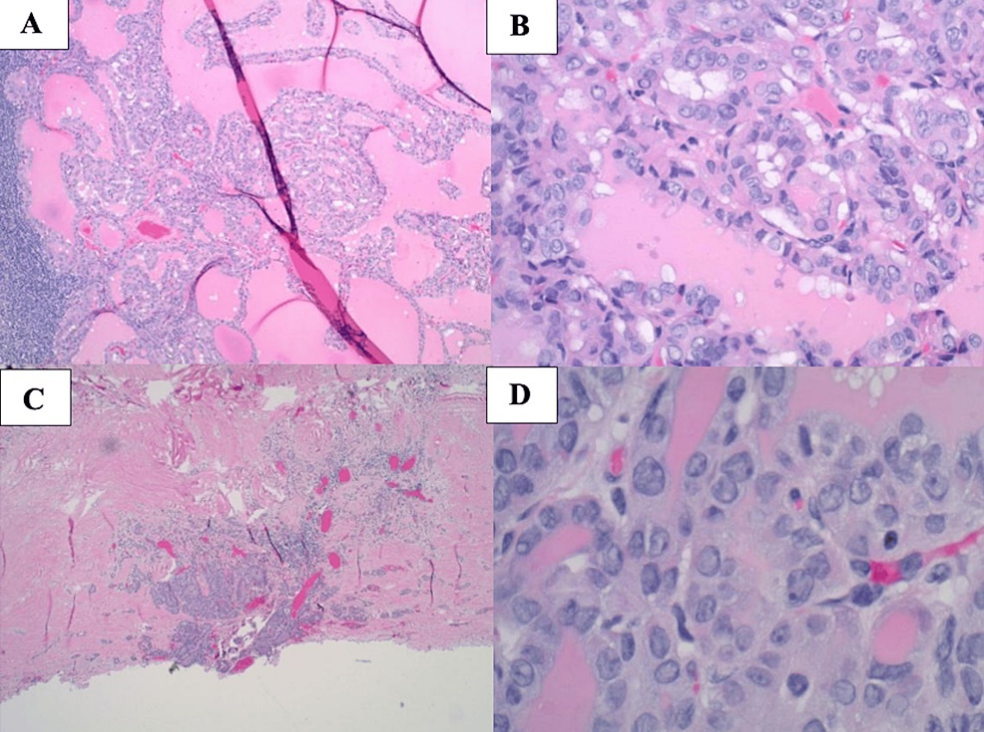
Lymph node dissection histological slides for Patient 1 A: Medium power image of specimen from right central compartment lymph node dissection. B: High power image of specimen from right central compartment lymph node dissection. C: Low power image of specimen from right neck level IV lymph node dissection. D: High power image of specimen from right neck level IV lymph node dissection.

**Figure 3:**
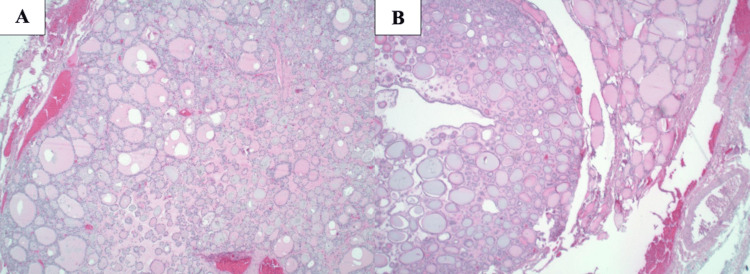
Histological slides of thyroid nodules for Patient 1 A: 14 mm isthmus nodule graded as TI-RADS 2 lesion. B: Right inferior pole 11 mm solid nodule graded as TI-RADS 3 lesion. TI-RADS: Thyroid Imaging, Reporting, and Data System

Final pathology demonstrated thyroid metastases in 1 of 8 lymph nodes in the right central nodal compartment and in 1 of 31 lymph nodes in the right neck (Figure 2A-2D). The only involved lateral neck lymph node was located at level IV, consistent with the preoperative imaging. Histological examination of the thyroid gland revealed the absence of malignant cells. Pathology was resubmitted for a second review with confirmation of previous results demonstrating no occult primary identified in the thyroid gland. Slides from the two thyroid nodules are also included in Figure 3A-3B demonstrating no evidence of carcinoma. Interestingly, the prelaryngeal mass was consistent with the thyroglossal duct cyst, which indicated abnormal embryologic development.

The patient had an unremarkable post-operative course. Parathyroid hormone levels were within normal limits. There were no ipsilateral shoulder range-of-motion deficits post-neck dissection. Post-operative thyroglobulin levels were 0.2-0.3. The patient underwent radioactive iodine ablation and made a successful recovery.

Patient 2

A 79-year-old male patient presented to the otolaryngology clinic with a painless and progressively growing mass in the left inferior parotid gland. Past medical history was significant for a left tonsillar squamous cell carcinoma treated with limited surgery (tonsillectomy only) and adjuvant radiation in the 1990s. The patient denied a history of fever, pain, dysphagia, dyspnea, weight loss, facial weakness, or dysphonia.

Physical exam demonstrated mass effect over the angle of the mandible. There was no palpable cervical adenopathy, tenderness, or trismus. The facial nerve exam was a 1/6 House-Brackmann bilaterally. A survey of the oral cavity mucosa and skin of the head and neck region revealed no obvious primary source. Of note, a left midline 2 mm smooth nodule was noted on the dorsal surface of the tongue. Given his head and neck cancer history, the differential diagnosis included new head and neck primary metastatic to the parotid gland, carcinoma unknown primary, and radiation-induced squamous cell carcinoma.

**Figure 4:**
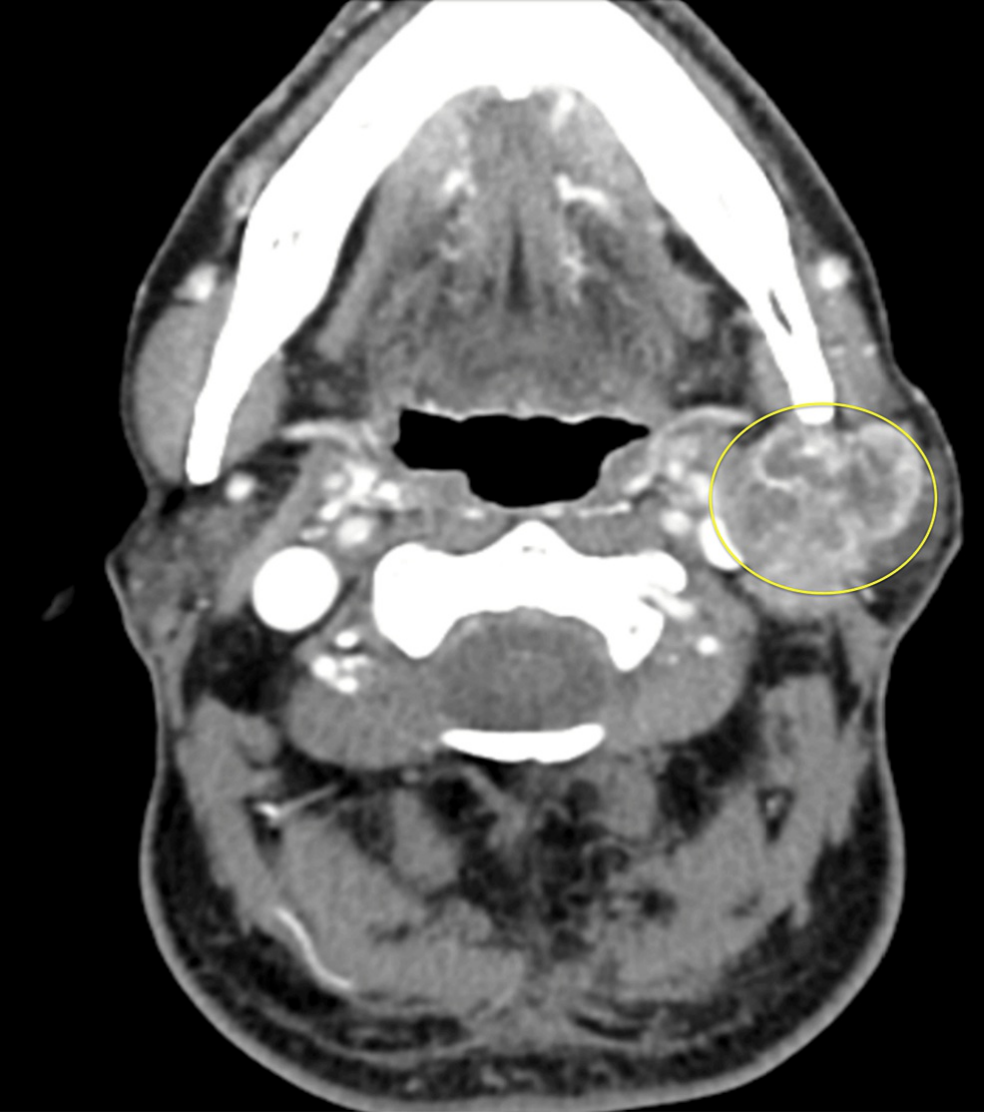
Axial-cut contrasted CT neck demonstrating a retromandibular, deep lobe parotid mass for Patient 2 Yellow circle: retromandibular, deep lobe parotid mass CT: computed tomography

Dedicated soft tissue computed tomography of the neck revealed a 4 cm solitary parotid mass with a retromandibular component concerning a deep lobe metastasis (Figure 4). No cervical adenopathy nor thyroid abnormality was noted bilaterally. Soft tissue ultrasound of the neck and thyroid were concordant. The thyroid did not have any nodules or cysts. Ultrasound-guided FNA of the parotid mass was consistent with PTC.

The patient underwent total thyroidectomy, left central nodal dissection, left modified radical neck dissection, left inferior superficial and deep lobe parotidectomy, and an excisional biopsy of the tongue. There was no evidence of thyroid carcinoma within the thyroid gland on pathological analysis or in any of the locoregional central, left level IIA, left level IIB, left level III, and left level IV lymph nodes. The surgical thyroid histology slides were sent for a second review, and the same conclusion was reached about an unknown primary thyroid carcinoma. Final pathology revealed that the 4.3 cm x 2.5 cm x 2.0 cm mass in the left deep parotid gland was positive for PTC. Interestingly, the mass of the right tongue was positive for moderately differentiated squamous cell carcinoma. Radioactive iodine was discussed, but the patient deferred. The patient continues to be biochemically and clinically free of thyroid carcinoma but developed a subsequent, likely radiation-induced squamous cell carcinoma of the tongue, for which they are undergoing treatment.

## Discussion

OTC is classified by the World Health Organization as an incidental pathology as it is often discovered inadvertently on imaging or on final pathology during operative interventions for other medical problems [9]. Of the five OTC subtypes, the fifth is one of the rarest variants. The different classifications of OTC are presented in Table 1 [4,5]. This subtype was first proposed by Liu et al., who described the presence of thyroid metastasis to nearby lymph nodes or organs even though the tissues in the thyroid gland are benign [4]. It is recommended that physicians conduct a comprehensive pathologic examination that ensures negative findings for cancerous growth in the thyroid gland. According to Liu et al., thyroid metastasis typically spreads to the central neck nodal compartment, where the involvement of these lymph nodes can be used as an indicator for locoregional spread [4]. As a result, central neck nodal dissections should be performed when there is suspicion of metastasis to nearby lymph nodes [4].

**Table 1:** Five different classifications of OTC OTC: occult thyroid carcinoma [4,5]

OTC classifications	Definitions
First type	Incidentally detecting OTCs after thyroidectomy for benign conditions
Second type	Incidental detection of OTC through fine needle biopsy or ultrasound examination
Third type	Prior to surgery, thyroid metastasis remains undetected, but the primary neoplasm becomes evident upon histological examination
Fourth type	Ectopic thyroid tissue that has metastasized or is symptomatic
Fifth type	Encompasses lymph node and organ metastasis despite the absence of primary thyroid gland cancer

Due to the unique presentation of OTC, this disease is often misdiagnosed, posing a significant risk due to its high likelihood to metastasize locoregionally and the chance for distant metastasis if it remains untreated. It is estimated that 20-90% of patients with papillary thyroid microcarcinoma will experience central neck lymph node metastasis [10]. However, the presence of central neck lymph node metastasis is not consistently detected on ultrasound, hence why performing FNA is another useful tool to confirm the presence of thyroid cancer metastasis [11]. Treatment of OTC may involve central neck lymph node dissections, either bilaterally or ipsilaterally, along with a total thyroidectomy to prevent further metastasis of the cancer and reduce recurrence rates [4,10,12]. Some studies have even suggested that performing a prophylactic central nodal dissection may allow for more precise staging and prevent tumor recurrence, while others have reported that this approach poses significant post-operative risks, such as injury to the recurrent laryngeal nerve or parathyroid glands [10,12]. Radioactive iodine ablation can also be used as supplemental adjuvant therapy following the removal of the metastatic malignant tissues in order to lower the risk of recurrence, although it does come with its own risks, such as sialadenitis and bone marrow suppression [11,13].

It is rare for PTC to metastasize to the parotid gland. One prospective study examined 15,780 patients and only identified three patients (0.019%) who experienced parotid metastases (PM) [14]. Similar to cervical lymph node metastasis, treatment for patients with PM may involve FNA biopsy, parotidectomy, total thyroidectomy, and modified radical neck dissection [14].

One possible explanation for thyroid cancer metastasis with undetectable malignancy within the thyroid gland is spontaneous regression of the malignancy within the thyroid gland. Host immunologic and apoptotic mechanisms may cause the malignancy within the thyroid gland to resorb [1,9]. Although rare, regression of tumors has been observed in renal cell carcinoma, cutaneous melanoma, and neuroblastoma [1,9]. A common sign that may point to tumor regression is histological fibrosis found in the thyroid gland [15]. Four patients described by Xu et al. presented with multifocal thyroid gland fibrosis, and one presented with unifocal thyroid gland fibrosis, even though they all had thyroid metastasis to the lymph nodes [1]. Fibrotic scars may further impair tumor development and promote its regression in the thyroid gland, which may explain the absence of malignancy within the thyroid gland on final pathology in rare cases of OTC [16]. Regarding the second case presented in this case report, another possible explanation for the lack of malignancy within the thyroid gland on final pathology could be that the radiation received 30 years prior may have resulted in significant fibrosis of the thyroid.

It is also possible that the tumor was overlooked during histopathologic examination, especially if the microcarcinoma in the thyroid gland was less than 3 mm in size [9]. Furthermore, ectopic thyroid tissue from other locations may cause metastasis to the cervical lymph nodes without originating in the thyroid gland [9]. Such may be the case for the first patient presented in this case report, where a thyroglossal duct cyst was present on the final pathology.

Genetic mutations, such as within the BRAF gene, have been observed in cases of OTC with cervical nodal metastasis, along with other types of thyroid carcinomas as well [1,17]. Genetic testing is an important tool for screening and detecting thyroid malignancies. Current research suggests that genetic mutations in the BRAF or RAS gene can significantly alter the pathogenesis of the MAPK, TRK, RET/PTC, and ALK pathways, which leads to the oncogenesis of the thyroid gland [18]. Some studies have suggested that BRAF mutations, especially BRAFV600E, are present in 60% of all PTC cases and are usually a predictive marker for occult metastasis [18,19]. The presence of these mutations on genetic screening in some studies has suggested indications for total thyroidectomy and even level VI neck dissection. Advancements in genetic testing, such as those from Foundation One or Veracyte, should be utilized, as they can identify targetable mutations, thereby guiding patients toward appropriate systemic, surgical, or salvage therapies [19,20]. In fact, one study demonstrated targetable BRAFV600E mutations in 83% of its PTC patients, highlighting the importance of genetic screenings in guiding treatment for thyroid malignancies [19].

Although the exact incidence and prevalence of metastatic thyroid carcinoma with an absent primary thyroid carcinoma are unknown, a 2017 paper suggested that only nine cases have been reported in the current literature [1]. Xu et al. claimed that even though there have been other studies that reported similar findings, it is unknown if they performed complete and thorough histologic examinations on the thyroid to rule out undetectable primary thyroid carcinoma [1]. It is certain that more cases have occurred since the publication of Xu et al., but the retrospective study further highlights the rare nature of this OTC subtype.

## Conclusions

Metastatic PTC may be misdiagnosed when there is no primary malignancy detected within the thyroid gland. Although rare, instances of cervical lymph node metastasis in OTC are typically discovered incidentally. Due to the rare nature of this phenomenon, it is important for medical providers to recognize possible metastatic locations for OTC and develop standardized diagnostic tools and treatment plans for patients with this condition.
